# MetaboLights: a resource evolving in response to the needs of its scientific community

**DOI:** 10.1093/nar/gkz1019

**Published:** 2019-11-06

**Authors:** Kenneth Haug, Keeva Cochrane, Venkata Chandrasekhar Nainala, Mark Williams, Jiakang Chang, Kalai Vanii Jayaseelan, Claire O’Donovan

**Affiliations:** European Molecular Biology Laboratory, European Bioinformatics Institute (EMBL-EBI), Wellcome Genome Campus, Hinxton, Cambridge CB10 1SD, UK

## Abstract

MetaboLights is a database for metabolomics studies, their raw experimental data and associated metadata. The database is cross-species and cross-technique and it covers metabolite structures and their reference spectra as well as their biological roles and locations. MetaboLights is the recommended metabolomics repository for a number of leading journals and ELIXIR, the European infrastructure for life science information. In this article, we describe the significant updates that we have made over the last two years to the resource to respond to the increasing amount and diversity of data being submitted by the metabolomics community. We refreshed the website and most importantly, our submission process was completely overhauled to enable us to deliver a far more user-friendly submission process and to facilitate the growing demand for reproducibility and integration with other ‘omics. Metabolomics resources and data are available under the EMBL-EBI’s Terms of Use via the web at https://www.ebi.ac.uk/metabolights and under Apache 2.0 at Github (https://github.com/EBI-Metabolights/).

## INTRODUCTION

Metabolomics is the systematic study of the small molecular metabolites in a cell, tissue, biofluid or cell culture media that are the tangible result of cellular processes or responses to an environmental stress. Collectively, these metabolites and their interactions within a biological system are known as the metabolome. Just as genomics is the study of DNA and genetic information within a cell, and transcriptomics is the study of RNA and differences in mRNA expression; metabolomics is the study of substrates and products of metabolism, which are influenced by both genetic and environmental factors. Metabolomics is a powerful approach because metabolites and their concentrations, unlike other ‘omics measures, directly reflect the underlying biochemical activity and state of cells / tissues. Because of this, metabolomics best represents the molecular phenotype. Metabolomics technologies yield many insights into basic biological research in areas such as systems biology and metabolic modelling, pharmaceutical research, nutrition and toxicology.

Our challenge is to capture the growing amount, depth and diversity of metabolomic information and to make it easily available and interpretable to our users and integrated with the wider ‘omics community. We describe the significant developments that we have made with a focus on how we are positioning MetaboLights (1) to address its increasing use in biosciences.

## PROGRESS AND DEVELOPMENTS

### Growth of submissions

**Figure 1.**
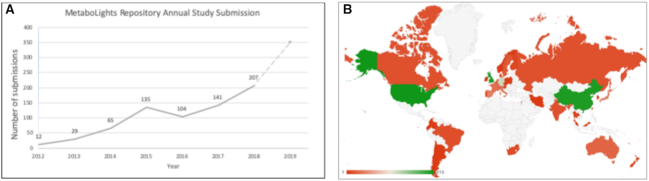
Growth and use of MetaboLights. (**A**) MetaboLights study submission rates per year (2019 total submissions estimated based on year to date). (**B**) Geographical distribution of submitted studies, including private studies (USA: 213, UK: 208, China: 207, Germany: 124, Spain: 44, Japan: 44, Italy: 36, France: 33, Netherlands: 31, Australia: 28, Belgium: 22, Sweden: 20).

The MetaboLights repository has continued to see consistent year on year growth since its first release in 2012, with a particularly notable exponential growth in the last year (Figure 1A). The user base is global with the USA, China and the UK being the top countries for study submissions (Figure 1B). At the time of writing, the database hosts over 500 publicly available studies with a further 140 awaiting public release due to publication requirements, and ∼250 in preparation. MetaboLights supports publications in a range of journals with a significant proportion not only in specialist metabolomics journals but also in journals from publication groups including Nature, Cell and PLOS. The studies in preparation reflect the fact that the database is evolving into a resource where data can be deposited throughout the experimental progress of a study and in the future, we plan to also provide pre-processing and analysis capabilities.

### New online guided submission and editor

**Figure 2.**
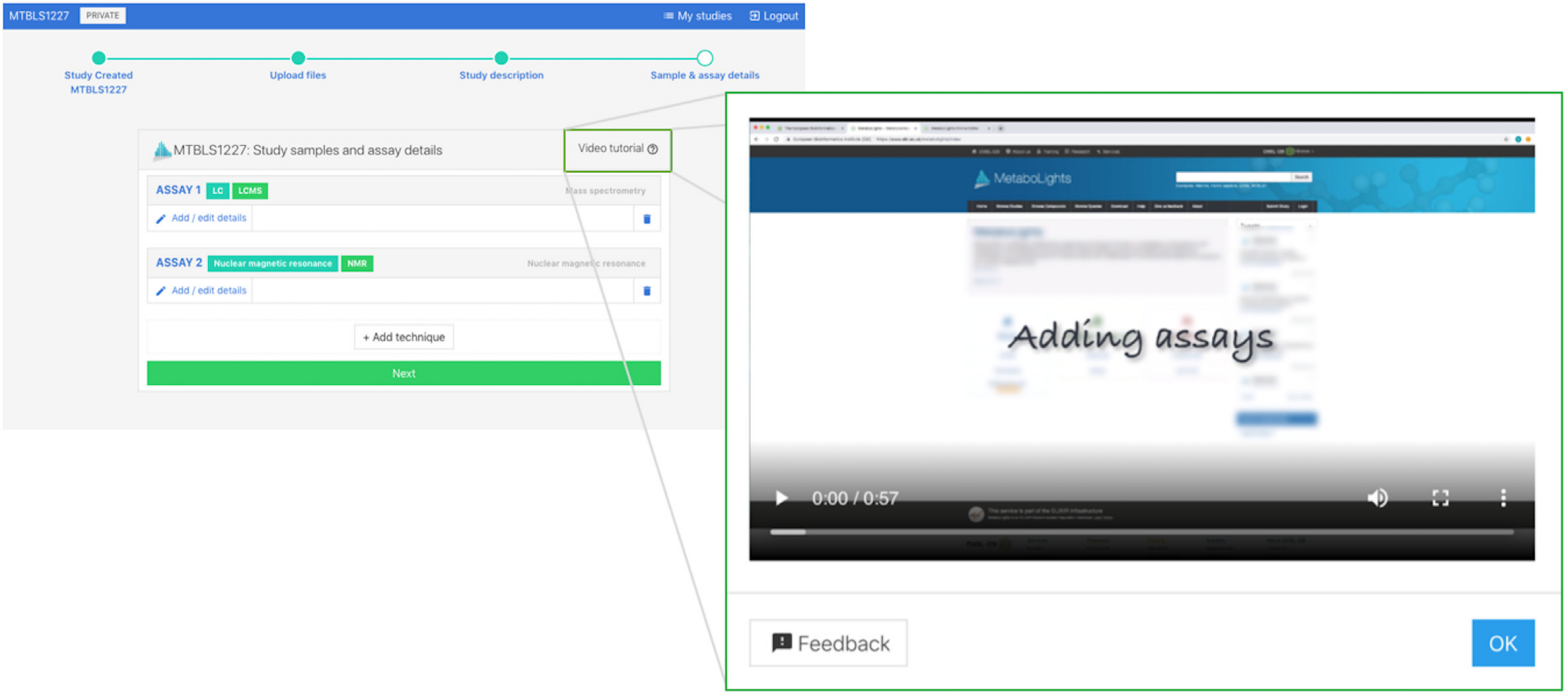
Submission process help.

The four stages in the MetaboLights submission and curation processes are: (i) *Submitted*, the user is adding all relevant raw data and information. (ii) *In curation*, the MetaboLights curation team is editing where required. (iii) *In review*, the study is ready to be shared with journals. (iv) *Public*, the study is publicly available. As part of our ongoing efforts to streamline and enhance the study submission and curation process, we have developed a new guided submission process to submit and edit studies online. This new submission tool (https://www.ebi.ac.uk/metabolights/editor/login) fully replaces our traditional desktop-based JAVA application (ISAcreator) (https://isa-tools.org/software-suite.html) while still using its functionalities relevant to metabolomics. The tool is also integrated with our resumable high-speed data transfer processes to conveniently enable large volumes of data transfer simultaneously while the user curates their study. The online submission tool is context-aware and guides submitters step-by-step through the process of describing the relevant experimental metadata, such as study characteristics, protocols, instrumentation and related factors. This enhances both the submitter experience and the accuracy and completeness of the study. To aid the submitter further, there are short video tutorials available at each stage of the submission process, see Figure 2.

Upon completion of the submission, the submitter is presented with a full study view to review and edit their study further should this be required. Going forward, we plan to extend this feature to facilitate ongoing studies/projects after the initial publication. Once the submitter is finished this initial stage, the MetaboLights curation team will start the final curation and quality control process and will interact with the submitter as appropriate to ensure high quality and comprehensive reporting of each study. Once this process is complete, the submitter will automatically receive instructions on how to safely allow direct read-only access to the study for the journals and their reviewers. The source code for the new submission tool (https://github.com/EBI-Metabolights/metabolights-editor) and the API ( https://github.com/EBI-Metabolights/MtblsWS-Py) are publicly available on GitHub.

In addition to the online submission process, we are actively working with the metabolomics community to provide solutions for larger scale resumable submission pipelines. A successful example of this is the recent development of a bespoke submission pipeline for metabolon (https://www.metabolon.com). Metabolon submits raw and metadata to MetaboLights on behalf of their clients who then complete the submission process with the online editor. Facilitated by the new MetaboLights RESTful API (https://www.ebi.ac.uk/metabolights/ws/api/spec.html#!/spec), we are now enabling programmatic submissions for Phenome centres and other large scale laboratories.

### Curation and content development

A MetaboLights study's discoverability and reusability is enhanced by mapping the metadata to the most relevant ontology resources. During the development of the online tool, the curators took the opportunity to review the content of all the studies and their related publications submitted since 2012. This highlighted how the metabolomics field is evolving, in both the science and the technology. For example, while there are 3400 species represented in the MetaboLights database, there has been an increasing deposition of human and mouse data which now constitutes 50% of the studies, reflecting the use of metabolomics in clinical applications. The techniques are also diversifying from variations of liquid chromatography (LC) and gas chromatography (GC) based mass spectrometry (MS) and nuclear magnetic resonance (NMR) to more recent developments in Image based MS and magnetic resonance. This review enabled us to align the metadata with the most relevant ontology resources (see Table 1) As a result, submitters are provided with the closest matching ontology linked vocabulary options within the new online editor. Where suitable terms are not available, submitters can make suggestions which are then reviewed by the curators. If the terms fall within the remit of an existing ontology, they are submitted to that resource. For more specialist terms, the curators are working to develop branches in reliable existing ontologies to serve the metabolomics community requirements. One existing and very successful collaboration with the ChEBI ontology team (2) illustrates how this kind of collaboration can evolve. ChEBI provides a uniform reference resource for metabolite nomenclature, associated chemical details and cross-omic referencing. All metabolites reported as part of a MetaboLights study submission are manually curated into the ChEBI ontology. In this way, the MetaboLights team has contributed over 4000 new metabolite entries to ChEBI together with experimental validation of over 20 000 more existing ChEBI entries. Curation of metabolites is a challenging and resource intensive activity and so we are developing a new automated submission pipeline between MetaboLights and ChEBI. This process will use publicly available compound resources like ChEBI, PubChem (3), ChemSpider (https://www.chemspider.com), Cactus (https://cactus.nci.nih.gove/chemical/structure), and OPSIN (4) to collate existing information including InChiKey, SMILES, InChi, IUPAC name, and synonym data and Classyfire (5) to assign ontological classes required for automatic inclusion in the ChEBI ontology. This will enable curators in both MetaboLights and ChEBI to focus on more expert curation and enable us to capture the complete data sets which benefits the community by enforcing the FAIR principles (https://www.force11.org/group/fairgroup/fairprinciples). All of this work has been informed by our collaboration with our longstanding colleagues in the ISA team at the University of Oxford (6) and the wider ISA commons community to ensure metabolomics continues to evolve in the wider ‘omics field.

**Table 1.** Examples of the most commonly referenced ontologies in MetaboLights

Area	Ontology
Species/Organism, Organism part	NCBI Taxonomy Database (7), World Register of Marine Species (WoRMS) (http://www.marinespecies.org), National Cancer Institute Thesaurus (NCIT (https://ncit.nci.nih.gov/ncitbrowser/), Brenda Tissue Ontology (BTO) (8)
Sample details (eg. disease state, treatment, timings)	National Cancer Institute Thesaurus (NCIT), Experimental Factor Ontology (EFO) (9), Plant Ontology (PO) (10), Units Ontology (UO) (11)
Instrumentation	MSIO (Metabolomics Standards Initiative Ontology), Chemical Methods Ontology (CHMO) (https://github.com/MSI-Metabolomics-Standards-Initiative/MSIO)
Metabolites	Chemical Entities of Biological Interest (ChEBI) (2)

### Website redesign

**Figure 3.**
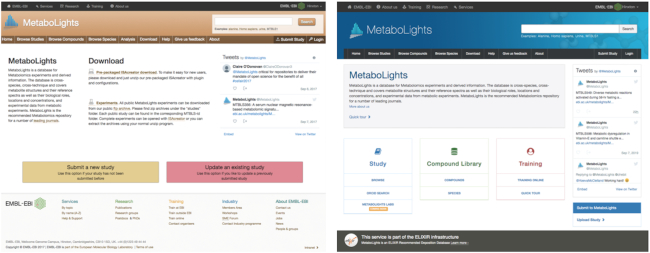
Website before (left) and after the redesign (right).

Over the last two years, the MetaboLights team has focused on updating the website for an improved user experience and study discoverability. Usability testing (UX) facilitated a more focused and intuitive view of the entire website and the data in the repository, see Figure 3. Newer JavaScript frameworks also ensure compatibility with various, portable, smaller screen sizes for the new website. The development will continue as we expand the functionality and services of the resource.

### Outreach and training

The MetaboLights team is dedicated to supporting the growth and development of the metabolomics field. A number of online resources are available through the EMBL-EBI online training portal (https://www.ebi.ac.uk/training/online/topic/metabolomics) which are very popular with thousands of hits per quarter. They include introductory lectures for metabolomics as well as webinars on specialist topics given by some of the leading experts in the field. The team has hosted an annual course ‘Introduction to Metabolomics Analysis’ for the last two years which has been very successful and oversubscribed. It teaches data handling, analysis and interpretation for newcomers to the field. The course offers a combination of lectures and hands on experience delivered in collaboration with experts in metabolomics from international laboratories. We are active participants in outreach at relevant international conferences, e.g. we were involved in four workshops at Metabolomics 2019 at the Hague, Netherlands.

## CONCLUSION

The MetaboLights team is committed to continually developing our resource and services to become a central hub for metabolomics related data and tools. This is a very exciting time in our field as more data is becoming available and the metadata is continually evolving. By redeveloping our submission processes, we have enabled our user community to make more data available as easily as possible. We will continue to work with the ISA commons community and we plan to develop a working area called MetaboLights Labs that will provide pre-processing and analysis capabilities to aid our submitters. We are also collaborating with our international colleagues, Metabolomics Workbench in the USA (https://www.metabolomicsworkbench.org) and MetaboBank in Japan (soon to be released at http://www.ddbj.nig.ac.jp) to both exchange data and to provide it in a variety of user-friendly ways for re-use and interpretation by the scientific community. Through these collaborations, we hope to deliver a key service and contribute to the further development of the field globally. We greatly value feedback from our user community. Please send your feedback and suggestions to metabolights-help@ebi.ac.uk or through the contact form at https://www.ebi.ac.uk/metabolights/contact.

## DATA AVAILABILITY

Metabolomics resources and data are available under the EMBL-EBI’s Terms of Use (https://www.ebi.ac.uk/about/terms-of-use) via the web at https://www.ebi.ac.uk/metabolights and under Apache 2.0 at Github (https://github.com/EBI-Metabolights/).

## Notes

Present address: Claire O’Donovan, European Molecular Biology Laboratory, European Bioinformatics Institute (EMBL-EBI), Wellcome Genome Campus, Hinxton, Cambridge CB10 1SD, UK.
